# Reliability and Validity of the HBSC Physical Activity Questionnaire in Japanese Adolescents

**DOI:** 10.3390/children12101360

**Published:** 2025-10-09

**Authors:** Chiaki Tanaka, Masashi Watanabe, Kan Oishi, John J. Reilly, Kojiro Ishii, Shigeho Tanaka

**Affiliations:** 1 Department of Human Nutrition, Tokyo Kasei Gakuin University, 2 Sanbancho, Chiyoda-ku 102-8341, Tokyo, Japan; 2 National Institute of Health and Nutrition, National Institutes of Biomedical Innovation, Health and Nutrition, Settsu 566-0002, Osaka, Japan; tanaka.shigeho@eiyo.ac.jp; 3 Institute of Nutrition Sciences, Kagawa Nutrition University, 3-9-21 Chiyoda, Sakado 350-0288, Saitama, Japan; 4 Faculty of Education, Ibaraki University, 2-1-1 Bunkyo, Mito 310-8512, Ibaraki, Japan; masashi.watanabe.1978@vc.ibaraki.ac.jp; 5 Faculty of Education, Saga University, 1 Honjo, Saga 840-8502, Saga, Japan; kaoishi@cc.saga-u.ac.jp; 6 Faculty of Health and Sports Science, Doshisha University, 1-3 Tatara Miyakodani, Kyotanabe-shi 610-0394, Kyoto, Japan; kishii@mail.doshisha.ac.jp; 7 Physical Activity for Health Group, Department of Psychological, Sciences and Health, University of Strathclyde, 16 Richmond Street, Glasgow G1 1XQ, UK; john.j.reilly@strath.ac.uk; 8 Faculty of Nutrition, Kagawa Nutrition University, 3-9-21 Chiyoda, Sakado 350-0288, Saitama, Japan

**Keywords:** exercise, self report, accelerometry, sex, adolescence

## Abstract

**Highlights:**

**What are the main findings?**

- The Japanese version of the WHO Health Behaviour in School-aged Children physical activity questionnaire (HBSC-J) had an acceptable reliability and validity for evaluating moderate-to-vigorous intensity physical activity (MVPA) in Japanese adolescents of both sexes.
- Duration, but not frequency, of vigorous physical activity as measured by the questionnaire was related to the accelerometer measurement in both sexes.

**What is the implication of the main finding?**

- The HBSC-J questionnaire is acceptable for evaluating daily MVPA in Japanese adolescents.
- An MVPA of 60 min per day as measured by an accelerometer corresponded to 3.6 days with 60 min per day measured by the questionnaire.

**Abstract:**

**Background/Objectives**: International physical activity (PA) questionnaires require a reliability and validity assessment in many countries to understand cross-cultural differences accurately. The current study examined the reliability and validity of the Japanese version of the WHO Health Behaviour in School-aged Children PA (HBSC-J) survey in adolescent students in Japan. **Methods**: The participants were 215 Japanese high school students. The HBSC-J was administered twice to measure reliability. The PA in the last week evaluated using the HBSC-J was compared with the PA evaluated using a triaxial accelerometer to measure the concurrent validity. **Results**: The intraclass correlation coefficients (ICCs) for reliability were 0.74 for the number of days, with 60 min/day or more of moderate-to-vigorous PA (MVPA). For the days with an MVPA of 60 min/day or more, ICCs were lower for girls (0.63 [0.49–0.74]) than boys (0.82 [0.75–0.87]). Positive correlations were observed between the accelerometry MVPA and the number of days, with at least 60 min/day of MVPA (r = 0.44). **Conclusions**: The HBSC-J questionnaire should be acceptable for evaluating MVPA in Japanese adolescents, with a reasonable reliability and validity.

## 1. Introduction

Concerning prevalences and trends of insufficient physical activity (PA) among adolescents aged 11–17 years have been recognized globally [1], but there are no national government PA surveys for Japanese adolescents. In the only survey by a local government, the Tokyo Metropolitan Survey of Physical Fitness, Physical Activity and Lifestyle 2011, pedometer-determined PA generally decreased with age across childhood and adolescence [2]. Organized sports participation among Japanese children and adolescents was highest among junior high school students (87% for boys, 69% for girls), but this rate dropped in high school, with a 20-point decrease for boys and 25-point decrease for girls [3]. Previous studies in other countries have also pointed out that a decline in participation in PA begins before or during early adolescence [4,5,6], becoming more pronounced from late adolescence through to young adulthood [7,8]. Therefore, it is important to monitor the current level of PA among late adolescents to properly understand levels of PA and inequalities in PA.

The largest international surveys for adolescents aged 11–17 years on health behaviors are the World Health Organization (WHO) surveys: the Health Behaviour among School-aged Children (HBSC) survey and the Global School-based Student Health Survey [1]. There is an urgent need to examine the validity and reliability of these survey instruments in different continents and cultures if national assessments of PA and cross-country comparisons are to be made with confidence [9]. A recent review pointed out that only a few studies have examined the reliability and validity of PA measurements derived from the HBSC questionnaire in junior high school and high school students [10]. The authors advocated for more reliability and validity studies across the entire adolescent age range. Moreover, a recent study from Vietnam not included in the above-mentioned review also reported an insufficient reporting of the reliability and validity of methods, particularly those focusing on late adolescence [11]. Although we confirmed the validity of the Japanese version of the WHO HBSC survey questionnaire (WHO HBSC-J) for primary and junior high school students from early to mid-adolescence (10–15 years old), the reliability and validity of this instrument in older adolescents (15–18 years old) is unclear [12]. Previous studies have generally found a high reliability of the HBSC PA question items [13,14]; however, the reliability might vary between countries/cultures, and the validity is less clear. The current study, therefore, examined the reliability and validity of the Japanese version of the HBSC-J in adolescents in Japan.

## 2. Materials and Methods

### 2.1. Participants

Students residing in urban areas of the Ibaraki prefecture and attending two public high schools were recruited for the current study (*n* = 231). Informed consent was obtained from all students and their parents. The study protocol was approved by the Human Research Ethics Committee of the affiliated university (Receipt Number: 22P0500). A total of 221 students (95.7% of those invited) and their parents agreed to participate in this study. This sample size was considered adequate to assess reliability and validity in both sexes separately [12]. Data on PA and anthropometric measurements were collected in June 2022.

### 2.2. Anthropometry

Stature and body weight were measured to the nearest 0.1 cm and 0.1 kg, respectively, with participants wearing clothing but no shoes. Net body weight was calculated by subtracting the estimated clothing weight from the measured body weight. Clothing consisted of standard shorts and a T-shirt, corresponding to a 0.5 kg deduction. Weight status was classified according to Japanese criteria for relative body weight, based on national reference data for Japanese adolescents [15]. Participants were categorized as normal weight, overweight/obese (+20% or more), or thin (−20% or less). Details of the equation are provided elsewhere [15].

### 2.3. Physical Activity Measurements

Habitual MVPA and vigorous PA (VPA) were evaluated using an Active style Pro accelerometer (HJA-750C; Omron Healthcare, Kyoto, Japan). Details on the accelerometer have been published elsewhere [16]. From the filtered three axis synthetic acceleration, one of three different equations was applied to predict metabolic equivalents (METs). A MET value was obtained in 10 s epochs, and the 1 min MET value was calculated as an average [16]. The accelerometer was worn on the right hip of the participant for 7–10 days. Participants were asked to wear the devices for all waking periods, except under special circumstances, such as dressing and bathing. Periods with 60 or more min of consecutive zero counts were considered to be non-wear time [17]. A day with 600 min (10 h) or more wear time was regarded as a valid day. Participants with data from at least two weekdays and one weekend day were included in the analysis [18]. The data obtained by the accelerometer were imported into a PC via BiLink (Omron Healthcare, Kyoto, Japan), and further data processing of the accelerometer was performed using the Excel macro program developed and distributed by the Japan Physical Activity Research Platform [19].

### 2.4. Self-Report Questionnaire

The HBSC study protocol 2013/14 survey questionnaire was used [20]. In the current study, the Japanese version of the HBSC questionnaire made through the back-translation process into English was used, as in our previous study [21]. HBSC is a WHO collaborative cross-national study that monitors the health and well-being of adolescents [22]. The questionnaire items of the HBSC have been revised in each survey round to reflect new and relevant issues. The HBSC examines both MVPA and VPA trends over time. Accordingly, three questionnaire items concerning MVPA and VPA were selected in the present study. The first question focuses on the number of days with 60 or more min/day of all types of activities undertaken in and out of school hours (walking to school, riding a bicycle, playing outside, walking fast, running, sports (e.g., swimming, dance, roller skating, skateboarding, surfing, soccer, basketball)), corresponding to MVPA [20]. The remaining two items—related to VPA—asked for frequency (per week) and total amount of time (duration per time) spent exercising vigorously outside school hours [20]. The first questionnaire completion corresponded to the last measurement date of the accelerometer, and then the second questionnaire completion took place 14 days later. Prior to data collection, one of the co-authors provided all participants with both a written document and an oral explanation of the study procedures. The same content was delivered to every participant to ensure consistency. During this session, students were informed that participation in the questionnaire was entirely voluntary and that there would be no disadvantage if they chose not to respond, although all students ultimately agreed to complete the questionnaire.

### 2.5. Statistical Analysis

For the accelerometer-derived data, daily averages of time spent in MVPA and VPA each day were calculated for each participant as the average number of weekday and weekend minutes spent in MVPA and VPA. The average weekly values were then calculated by weighting for five weekdays and two weekend days as follows: weighted weekly average = [(weekday average × 5) + (weekend average × 2)]/7}.

To evaluate the test–retest reliability of the PA questions, intraclass correlation coefficients (ICCs) were calculated. A 95% confidence interval (CI) was used to express the variability in the ICCs. ICCs above 0.90 are considered excellent; 0.75–0.90 is good; 0.50–0.75 is moderate; and below 0.50 is classified as poor agreement [23].

The validity of the PA questionnaire was assessed using partial correlation analyses, adjusting for age, sex, and relative weight. These correlations indicate the association between self-reported PA metrics (e.g., number of days, frequency, duration) and accelerometer-measured MVPA or VPA. To explore sex differences in age, anthropometric data, and PA levels, paired *t*-tests were conducted. The Shapiro–Wilk test was used to assess normality, considering the relatively small sample size in the present study. For non-normally distributed variables, Mann–Whitney U tests and Chi-square tests were applied to compare PA levels between girls and boys. Characteristics of participants excluded from the validation study were examined by comparing them with included participants, adjusting for sex using an analysis of covariance (ANCOVA). To examine the relationship between days with MVPA of 60 min per day or more measured by the questionnaire and MVPA minutes per day measured by the accelerometer, a regression analysis was employed. All statistical analyses were conducted using SPSS version 28.0J (IBM, Japan, Tokyo). A *p*-value of ≤0.05 was considered statistically significant.

## 3. Results

### 3.1. Participant Characteristics

The initial participant pool included 221 students. After excluding 6 individuals who did not complete the questionnaire, 215 participants remained for the reliability analysis. One girl and nine boys (4.7%) were classified as overweight/obese, while two girls and four boys (2.8%) were classified as underweight. For the validation analysis, 67 students were excluded due to insufficient accelerometer data, resulting in a final sample of 154. One girl and five boys (3.9%) were classified as overweight/obese, while two girls (1.3%) were classified as underweight. After adjusting for sex using an ANCOVA, no significant differences were observed in age, height, weight, or relative body weight between the 67 excluded individuals and the 154 participants.

Descriptive statistics for participant characteristics, accelerometer-measured MVPA and VPA, and self-reported PA (frequency and duration) are presented in Table 1, Table 2, Table 3 and Table 4. On average, participants wore the accelerometer for 6.2 days, with a mean daily wear time of 16.0 h. Weight, relative weight, and the MVPA and VPA measured by the accelerometer in girls were all significantly lower than in the boys.

### 3.2. Reliability and Validity of the PA Questions in the HBSC-J Questionnaire

The reliability of the MVPA and VPA questionnaire items was moderate, with ICCs of 0.74 for days achieving ≥60 min of MVPA, 0.71 for frequency, and 0.64 for duration (Table 5). Notably, ICC values for girls were slightly lower than those for boys.

The average number of days with 60 min per day or more was 3.8 ± 2.1 days for girls and 4.7 ± 2.0 days for boys. Significant positive correlations were observed between the MVPA measured by accelerometry and the number of days with at least 60 min per day of MVPA recorded by the HBSC-J (r = 0.44 for all, *p* < 0.001; r = 0.41 for girls, *p* < 0.001; r = 0.48 for boys, *p* < 0.001) (Table 6). Significant negative correlations (r = −0.19 for all, *p* = 0.017; r = −0.26 for girls, *p* = 0.021) were obtained between the VPA measured by accelerometry and recorded by the HBSC-J question on VPA frequency. Significant positive correlations (r = 0.24 for all, *p* = 0.003; r = 0.40 for girls, *p* < 0.001; r = 0.24 for boys, *p* = 0.040) were observed between the VPA measured by accelerometer and the HBSC-J question on VPA duration.

**Figure 1:**
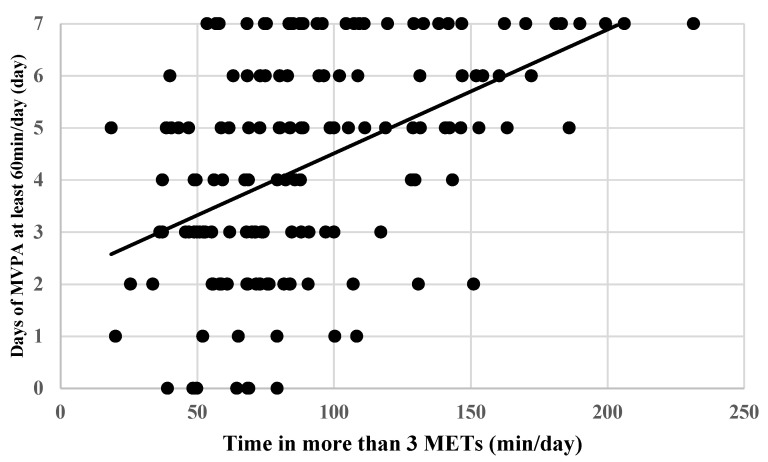
Correlation between days with moderate-to-vigorous physical activity of 60 min per day or more recorded by the questionnaire and moderate-to-vigorous physical activity minutes per day measured by the accelerometer. Abbreviations: METs: metabolic equivalents; MVPA: moderate-to-vigorous physical activity.

The regression equation between days with an MVPA of 60 min per day or more recorded by the question and MVPA minutes per day measured by the accelerometer was obtained (Figure 1).

Y (days with MVPA of 60 min per day or more) = 2.1 + 0.024 × MVPA (min/day)

As a result, an MVPA of 60 min per day measured by the accelerometer corresponded to 3.6 days with 60 min per day recorded by the questionnaire.

## 4. Discussion

Physical activity is such an important determinant of adolescent physical and mental health that much effort has gone into understanding between-country differences in PA [1,24], but doing so depends on the use of reliable and valid measurement instruments which can be used across many different countries/cultures. The aim of the current study was therefore to examine the reliability and validity of the HBSC-J questionnaire in late adolescents. Overall, the self-reported HBSC-J questions on MVPA had a moderate reliability and correlated significantly with the accelerometer-measured MVPA in both girls and boys. Our findings suggest that cross-cultural comparisons of adolescent levels of PA using the HBSC questionnaire would be justifiable. While the reported VPA items were moderately reliable for the frequency of VPA and weakly correlated for the duration of VPA with accelerometer data in terms of the VPA minutes per day of students, both girls and boys, the correlations for VPA frequency were significantly negative. The finding of cross-cultural validity for MVPA measurement might provide encouragement that the HBSC questionnaire is fairly generalizable across different cultures.

The test–retest reliability was moderate for MVPA and VPA items in the whole sample, with a better reliability for the MVPA item in boys than in girls. MVPA and VPA evaluated by the accelerometer for girls was lower than those for boys. Reasons for these sex differences are unclear, but girls might have had fewer opportunities to engage in VPA, making it more difficult to recall, and a lower engagement in VPA might make recall more challenging [25]. To our knowledge, no previous study evaluated the test–retest reliability of the MVPA item for high school students. Rangul et al. [14] reported that the ICCs (8–12 days apart) were 0.71 for VPA frequency and 0.73 for VPA duration in adolescents aged 13–18 years in Norway. For the frequency question, different ICCs were obtained between girls (0.87) and boys (0.59). Booth et al. [13] showed that VPA frequency or duration values of kappa (14 days apart) were 0.57 (95% CI: 0.34–0.80) or 0.52 (95% CI: 0.29–0.75) for girls and 0.60 (95% CI: 0.48–0.72) or 0.58 (95% CI: 0.47–0.70) for high school boys in year 10 (mean age 15.1 years) from Australia. Although the time between the two tests has an influence on reliability [26,27], the time between test and retest was similar between previous studies and the current study. In general, the reliability of VPA items was broadly comparable to studies among Australian and Norwegian high school students [13,14]. However, previous studies found that the VPA items tended to be more reliable for girls than for boys [13,14]. In our study, the MVPA item tended to be more reliable for boys, while there were no sex differences in VPA items that asked about the frequency and duration of exercise. The duration, frequency, and intensity of organized sports or active transportation might be more easily recalled than unplanned or occasional MVPA or VPA in daily activities. In a national survey in Japan, 67% of high school students participated in organized sports, compared with 44% of girls [3]. The Sasakawa sports foundation national survey in 2015 reported that 68% of 15–17-year-olds regularly commuted actively (walking or cycling) to school and almost 80% of boys used active transport [28]. A previous study has reported that high active commuting rates are a cultural feature of Japan compared to other countries located on six continents [24]. For these reasons, the MVPA item tended to be more reliable for boys in the current study. Negative correlations between VPA frequency and the accelerometer data were obtained in both girls and boys (r = −0.26; r = −0.23). The reasons for this observation cannot be determined with a high confidence by the present study, and detailed speculation might be inappropriate. However, we note that, in both sexes, the frequency of reported VPA was very low, and the time spent in VPA averaged <3 min/day in girls and <10 min/day in boys (Table 4). Activities which are both rare and brief might be challenging to report accurately, and this might have contributed to the negative correlation between self-reported VPA frequency and accelerometer-measured VPA in both girls and boys.

A recent review pointed out that only a few studies have examined the validity of the PA measure derived from the HBSC questionnaire [10]. For high school students, Booth et al. [13] investigated the relationships between the frequency and duration of VPA questions for the original English version of the HBSC survey and used aerobic fitness to assess the discriminant validity for high school students in Australia. The brief self-report questions on participation in VPA appeared to have an acceptable validity in high school students [14]. Rangul et al. [14] found that the validity of the HBSC questionnaire measured against VO_2_ peak was significant for both sexes (r = 0.39 for frequency; r = 0.33 for duration) and girls (r = 0.55 for frequency; r = 0.41 for duration), but not for boys. These previous validation studies for high school students used aerobic fitness rather than physical activity levels as the validity criterion. This methodological difference prevents a direct comparison with the results of this study.

The questionnaire showed no correlation with the physical activity level and total energy expenditure predicted by the ActiReg (PreMed AS, Oslo, Norway) activity monitor [14]. Accelerometry is a widely used method to assess the concurrent validity of PA questionnaires [29,30,31]. The validity of the HBSC for high school students has not been reported against the duration of MVPA or VPA. Chinapaw et al. [29] reported a systematic review for the validity and reliability of potential PA questionnaires in adolescents (mean age < 18 y). In 12–18-year-olds, the correlations between PA questionnaires and accelerometers were from r = 0.12 (Child Heart and Health Study in England Questionnaire) to r = 0.49 (PA Questionnaire for Adolescents). The partial correlation coefficients in the current study between MVPA measured by accelerometry and the HBSC-J questions in both girls and boys were similar to those of many previous studies.

The present study developed an equation relating the achievement of 60 min/day of MVPA on average to the number of days per week it was reported as being achieved: the accelerometer-measured 60 min of daily MVPA corresponded to 3.6 days per week with 60 min of MVPA recorded in the questionnaire, indicating that questionnaire-based MVPA assessment did not fully reflect the accelerometer-measured MVPA. Applying our equation, 60 min of daily MVPA corresponds to 204 min of MVPA using the HBSC-J. This would serve as a practical target for achievement when using the HBSC-J.

There are limitations in the interpretation of the results of the current study. First, the participants in the current study were not nationally representative of Japanese adolescents, though for a methodological study this may be considered a minor weakness. Second, our participants were recruited from only two schools in a single urban area. Thus, the results of the current study may only be applicable to Japanese students in urban areas. In rural areas, declining birthrates have led to school closures. As a result, students often face limitations such as access to only a few types of sports or being unable to form teams due to insufficient numbers. Moreover, students are increasingly reliant on school buses or being driven by their parents in private vehicles for commuting to school. Therefore, the lifestyle of high school students in rural areas may differ from that of their urban counterparts. Future studies and research are needed in rural areas. For a higher generalizability, reliability and validity should be examined in various types of schools and areas, including rural areas. Third, for the validation analysis, the exclusion rate (30%) was high. This was due to the influence of contact sports such as judo and swimming, as well as weekend competitions during which the participants were unable to wear accelerometers in accordance with the competition rules. Finally, the accelerometer’s insensitivity to activities like cycling and swimming may have underestimated MVPA.

## 5. Conclusions

The current study found that the WHO HBSC-J questionnaire had an acceptable reliability, and the validity was moderate for evaluating MVPA in Japanese high school girls and boys. The reported duration of VPA also reflected the average VPA time, but weakly and with a lower validity than MVPA reports made by both girls and boys. Physical inactivity in populations is usually operationalized as the prevalence of not meeting the recommended levels of MVPA. Therefore, the effectiveness of MVPA assessment demonstrated in the present study may provide valuable tools and insights for shaping future national and regional health policies in Japan. This finding may also serve as a valuable contribution to the progress of international comparative PA research.

## Figures and Tables

**Table 1:** Descriptive characteristics of participants of the reliability study.

	All (*n* = 215)			Girls (*n* = 93)			Boys (*n* = 122)		
	Mean	±	SD	Mean	±	SD	Mean	±	SD
Age (years)	16.7	±	0.8	16.8	±	0.8	16.7	±	0.9
Height (cm) *	165.1	±	8.3	158.2	±	5.4	170.4	±	5.8
Weight (kg) *	55.9	±	8.7	50.2	±	5.7	60.2	±	8.1
Body Mass Index (kg/m^2^) *	20.5	±	2.4	20.1	±	2.0	20.7	±	2.6
Relative Weight (%) *	−1.3	±	11.8	−3.5	±	9.9	0.4	±	12.9

Abbreviations: SD: standard deviation. Bolded items with * indicate a significant sex difference (*p* < 0.05).

**Table 2:** Physical activity profile of participants in the reliability study.

Daily Physical Activity by Questionnaire	At Baseline						Follow-Up					
	All (*n* = 215)		Girls (*n* = 93)		Boys (*n* = 122)		All (*n* = 215)		Girls (*n* = 93)		Boys (*n* = 122)	
	*n*	(%)	*n*	(%)	*n*	(%)	*n*	(%)	*n*	(%)	*n*	(%)
Days of MVPA at least 60 min/day												
0 (day/week)	14	(6.5)	7	(7.5)	7	(5.7)	16	(7.4)	6	(6.5)	10	(8.2)
1 (day/week)	10	(4.7)	7	(7.5)	3	(2.5)	8	(3.7)	4	(4.3)	4	(3.3)
2 (day/week)	32	(14.9)	18	(19.4)	14	(11.5)	29	(13.5)	20	(21.5)	9	(7.4)
3 (day/week)	33	(15.3)	10	(10.8)	23	(18.9)	36	(16.7)	15	(16.1)	21	(17.2)
4 (day/week)	21	(9.8)	11	(11.8)	10	(8.2)	18	(8.4)	7	(7.5)	11	(9.0)
5 (day/week)	36	(16.7)	19	(20.4)	17	(13.9)	32	(14.9)	16	(17.2)	16	(13.1)
6 (day/week)	26	(12.1)	8	(8.6)	18	(14.8)	30	(14.0)	10	(10.8)	20	(16.4)
7 (day/week)	43	(20.0)	13	(14.0)	30	(24.6)	46	(21.4)	15	(16.1)	31	(25.4)
Frequency of VPA by questionnaire												
Every day	24	(11.2)	15	(16.1)	9	(7.4)	24	(11.2)	16	(17.2)	8	(6.6)
4 to 6 times a week	10	(4.7)	7	(7.5)	3	(2.5)	11	(5.1)	6	(6.5)	5	(4.1)
2 to 3 times a week	11	(5.1)	4	(4.3)	7	(5.7)	12	(5.6)	2	(2.2)	10	(8.2)
Once a week	32	(14.9)	16	(17.2)	16	(13.1)	22	(10.2)	8	(8.6)	14	(11.5)
Once a month	42	(19.5)	16	(17.2)	26	(21.3)	43	(20.0)	24	(25.8)	19	(15.6)
Less than once a month	60	(27.9)	26	(28.0)	34	(27.9)	72	(33.5)	27	(29.0)	45	(36.9)
Never	36	(16.7)	9	(9.7)	27	(22.1)	31	(14.4)	10	(10.8)	21	(17.2)
Duration of VPA by questionnaire												
None	41	(19.1)	26	(28.0)	15	(12.3)	40	(18.6)	21	(22.6)	19	(15.6)
About half an hour	44	(20.5)	23	(24.7)	21	(17.2)	30	(14.0)	11	(11.8)	19	(15.6)
About 1 h	38	(17.7)	11	(11.8)	27	(22.1)	40	(18.6)	20	(21.5)	20	(16.4)
About 2 to 3 h	37	(17.2)	13	(14.0)	24	(19.7)	40	(18.6)	20	(21.5)	20	(16.4)
About 4 to 6 h	24	(11.2)	10	(10.8)	14	(11.5)	25	(11.6)	12	(12.9)	13	(10.7)
About 7 h or more	31	(14.4)	10	(10.8)	21	(17.2)	40	(18.6)	9	(9.7)	31	(25.4)

Abbreviations: MVPA: moderate-to-vigorous physical activity; VPA: vigorous physical activity.

**Table 3:** Descriptive characteristics of participants in the validity study.

	All (*n* = 154)			Girls (*n* = 78)			Boys (*n* = 76)		
	Mean	±	SD	Mean	±	SD	Mean	±	SD
Age (years)	16.8	±	0.9	16.8	±	0.9	16.7	±	0.9
Height (cm) *	164.2	±	8.4	158.0	±	5.4	170.6	±	5.7
Weight (kg) *	55.4	±	8.5	50.2	±	5.5	60.8	±	7.7
Body Mass Index (kg/m^2^) *	20.5	±	2.4	20.1	±	2.0	20.9	±	2.6
Relative Weight (%) *	−1.3	±	11.4	−3.5	±	9.6	1.0	±	12.8

Abbreviations: SD: standard deviation. Bolded items with * indicate a significant sex difference (*p* < 0.05).

**Table 4:** Physical activity profile of participants in the validity study.

Daily Physical Activity by Accelerometer	All (*n* = 154)			Girls (*n* = 78)			Boys (*n* = 76)		
	Mean	±	SD	Mean	±	SD	Mean	±	SD
Time in more than 3 METs (min/day) *	89.9	±	41.0	77.0	±	29.3	103.2	±	47.0
Time in more than 6 METs (min/day) *	5.5	±	8.1	2.5	±	3.1	8.7	±	10.2
Daily physical activity by questionnaire									
Days of MVPA at least 60 min/day	*n*		(%)	*n*		(%)	*n*		(%)
0 (day/week)	7		(4.5)	5		(6.4)	2		(2.6)
1 (day/week)	6		(3.9)	5		(6.4)	1		(1.3)
2 (day/week)	24		(15.6)	16		(20.5)	8		(10.5)
3 (day/week)	25		(16.2)	10		(12.8)	15		(19.7)
4 (day/week)	14		(9.1)	9		(11.5)	5		(6.6)
5 (day/week)	28		(18.2)	14		(17.9)	14		(18.4)
6 (day/week)	17		(11.0)	6		(7.7)	11		(14.5)
7 (day/week)	33		(21.4)	13		(16.7)	20		(26.3)
Frequency of VPA by questionnaire									
Every day	17		(11.0)	13	(16.7)		4		(5.3)
4 to 6 times a week	10		(6.5)	8	(10.3)		2		(2.6)
2 to 3 times a week	8		(5.2)	4	(5.1)		4		(5.3)
Once a week	27		(17.5)	14	(17.9)		13		(17.1)
Once a month	28		(18.2)	13	(16.7)		15		(19.7)
Less than once a month	41		(26.6)	20	(25.6)		21		(27.6)
Never	23		(14.9)	6	(7.7)		17		(22.4)
Duration of VPA by questionnaire									
None	35		(22.7)	25	(32.1)		10		(13.2)
About half an hour	30		(19.5)	17	(21.8)		13		(17.1)
About 1 h	26		(16.9)	8	(10.3)		18		(23.7)
About 2 to 3 h	26		(16.9)	11	(14.1)		15		(19.7)
About 4 to 6 h	17		(11.0)	9	(11.5)		8		(10.5)
About 7 h or more	20		(13.0)	8	(10.3)		12		(15.8)

Abbreviations: SD: standard deviation. Bolded items with * indicate a significant sex difference (*p* < 0.05). METs: metabolic equivalents; MVPA: moderate-to-vigorous physical activity; VPA: vigorous physical activity.

**Table 5:** Test–retest reliability based on intraclass correlation coefficients (ICCs) for the Japanese version of the WHO HBSC questionnaire.

	All (*n* = 215)	Girls (*n* = 93)	Boys (*n* = 122)
	ICC [95% CI]	ICC [95% CI]	ICC [95% CI]
Days of at least 60 min/day MVPA (days/week)	0.74 [0.68–0.80]	0.63 [0.49–0.74]	0.82 [0.75–0.87]
Frequency of VPA (days/week)	0.71 [0.64–0.77]	0.72 [0.60–0.80]	0.69 [0.58–0.77]
Duration of VPA (hours/week)	0.64 [0.55–0.83]	0.64 [0.50–0.75]	0.62 [0.50–0.72]

Abbreviations: ICC: single measure intraclass correlation coefficient; CI: confidence interval; MVPA: moderate-to-vigorous physical activity; VPA: vigorous physical activity.

**Table 6:** Partial correlations between self-reported and accelerometry-recorded physical activity levels.

By Accelerometer (min/day)	All (*n* = 154)		Girls (*n* = 78)		Boys (*n* = 76)	
	MVPA [95% CI]	VPA [95% CI]	MVPA [95% CI]	VPA [95% CI]	MVPA [95% CI]	VPA [95% CI]
Days of at least 60 min/day MVPA (days/week)	0.44 [0.30–0.56]		0.41 [0.21–0.58]		0.48 [0.28–0.64]	
	*p* < 0.001		*p* < 0.001		*p* < 0.001	
Frequency of VPA (days/week)		−0.19 [−0.34–−0.03]		−0.26 [−0.46–−0.04]		−0.23 [−0.44–0.00]
		*p* = 0.017		*p* = 0.021		*p* = 0.053
Duration of VPA (hours/week)		0.24 [0.08–0.38]		0.40 [0.19–0.57]		0.24 [0.02–0.43]
		*p* = 0.003		*p* < 0.001		*p* = 0.040

Abbreviations: MVPA: moderate-to-vigorous physical activity; VPA: vigorous physical activity; CI: confidence interval. A partial correlation adjusted for sex, age, and relative weight between the answers to the survey questions and objectively measured moderate-to-vigorous physical activity or vigorous physical activity recorded by an accelerometer.

## Data Availability

The participants of this study did not give written consent for their data to be shared publicly, so due to the sensitive nature of the research supporting data is not available.

## Abbreviations

The following abbreviations are used in this manuscript:

PA	Physical activity
HBSC	The WHO Health Behaviour in School-aged Children physical activity
HBSC-J	The Japanese version of the WHO Health Behaviour in School-aged Children physical activity
ICCs	Intraclass correlation coefficients
MVPA	Moderate-to-vigorous physical activity
WHO	World Health Organization
VPA	Vigorous physical activity
METs	Metabolic equivalents
CI	Confidence interval
SD	Standard deviation
